# Cost effectiveness of nusinersen for patients with infantile-onset spinal muscular atrophy in US

**DOI:** 10.1186/s12962-020-00234-8

**Published:** 2020-10-06

**Authors:** Praveen Thokala, Matt Stevenson, Varun M. Kumar, Shijie Ren, Alexandra G. Ellis, Richard H. Chapman

**Affiliations:** 1 School of Health and Related Research, University of Sheffield, Sheffield, UK; 2 Previously At Institute for Clinical and Economic Review (ICER), Boston, MA USA; 3 Institute for Clinical and Economic Review (ICER), Boston, MA USA

**Keywords:** Cost-effectiveness, Spinal muscular atrophy (SMA), Nusinersen

## Abstract

**Background:**

Patients with infantile-onset spinal muscular atrophy (SMA), a rare, genetic neuromuscular disease, do not achieve key motor function milestones (e.g., sitting) and have short life expectancy in the absence of treatment. Nusinersen is a disease-modifying therapy for patients with SMA.

**Objective:**

The aim of this study was to estimate the cost-effectiveness of nusinersen compared to best supportive care (BSC) in patients diagnosed with infantile-onset SMA in the US.

**Methods:**

A de novo economic model was developed with the following health states: “permanent ventilation”, “not sitting”, “sitting”, “walking”, and “death”. Short-term data were sourced from the pivotal clinical trials and studies of nusinersen (ENDEAR and SHINE). Motor function milestones achieved at the end of follow-up in the clinical trials were assumed to be sustained until death. Mortality risks were based on survival modelling of relevant published Kaplan–Meier data. Costs, life years (LYs), and quality-adjusted life years (QALYs) were discounted at 3% per annum, and the analyses were performed from a US health care sector perspective. Scenario analyses and sensitivity analyses were conducted to assess the robustness of the results to key parameters.

**Results:**

In our base-case analysis, nusinersen treatment achieves greater QALYs and more LYs (3.24 and 7.64, respectively) compared with BSC (0.46 QALYs and 2.40 LYs, respectively), resulting in an incremental cost per QALY gained of approximately $1,112,000 and an incremental cost per LY gained of $590,000 for nusinersen compared to BSC. The incremental cost effectiveness ratios did not fall below $990,000 per QALY gained in scenario and sensitivity analyses. Results were most sensitive to the length of survival, background health care costs, and utility in the “not sitting” and “sitting” health states.

**Conclusions:**

The estimated incremental cost-effectiveness of nusinersen from a US health care sector perspective exceeded traditional cost-effectiveness thresholds. Cost-effectiveness was dependent on assumptions made regarding survival, costs, utilities, and whether the motor function milestones were sustained over lifetime. Given the relatively short-term effectiveness data available for the treatment, a registry to collect long-term data of infantile-onset SMA patients is recommended.

## Background

Spinal muscular atrophy (SMA) is a rare, genetic neuromuscular disease with the most severe case of infantile-onset SMA (Type I SMA) affecting infants and young children. [1, 2] In the United States (US), SMA incidence is approximately one in 10,000 live births or about 500 new SMA cases per year, of which infantile-onset SMA represents approximately 60% of cases. [3]

Patients with infantile-onset SMA do not achieve key motor function milestones (e.g., sitting) and have short life-expectancy in the absence of treatment. Historically, life expectancy in these infantile-onset SMA patients was less than 2 years and many infants eventually require permanent ventilation. To maintain mobility and function as long as possible, multidisciplinary, supportive care including respiratory, nutritional, gastrointestinal, orthopedic, and other support is needed. [4–6] However, supportive care does not modify disease progression and patients may be entirely dependent on family members and caregivers.

In December 2016, the Food and Drug Administration (FDA) approved nusinersen (Spinraza®, Biogen Idec), a disease-modifying therapy, for the treatment of SMA. [7] It is administered via intrathecal injection (into the fluid surrounding the spinal cord) with four loading doses (day 0, day 14, day 28, and day 63) and maintenance doses every 4 months thereafter. Nusinersen has been studied in patients with or likely to develop SMA [8–10], with several studies ongoing [11–14].

There are two clinical trials of nusinersen in infantile-onset SMA, including a phase II, open-label, dose-escalation study (CS3A) [8] and a randomized controlled trial with sham control (ENDEAR). [9] For ENDEAR, an interim analysis showed statistical superiority of Hammersmith Infant Neurological Examination-“Methods Section” (HINE-2) responders favoring nusinersen and the study was subsequently terminated prior to the planned 13 month follow up. However, longer-term results are available for infants in ENDEAR who enrolled in the single-arm open label extension (OLE) study SHINE. [15].

The aim of this paper is to present the cost-effectiveness of nusinersen for treatment of patients with infantile-onset SMA in the US. Section two (Methods) describes the model structure, key assumptions, input data, and analyses. Section three (Results) presents the results of base-case, scenario, one-way and probabilistic sensitivity analyses. Section four (Discussion) highlights the key points, model limitations and comparison to other models. The final section presents the conclusions.

## Methods

A de novo model was developed in Microsoft Office Excel 2016, to estimate the lifetime cost-effectiveness of nusinersen compared to best supportive care (BSC) for patients with infantile-onset SMA, from the US health care sector perspective. Costs, life years (LYs), and quality-adjusted life years (QALYs) were discounted at 3% per annum. A modified societal perspective scenario analysis was also performed, including patient-centric societal costs (i.e., non-medical costs) and productivity gains, along with patient QALYs and health care costs. This model has also been used in the Institute for Clinical and Economic Review (ICER) evaluation of nusinersen and onasemnogene abeparvovec-xioi (Zolgensma®, Avexis) for SMA. [16] Input was sought from the manufacturers, patient groups, health economists and clinical experts throughout the model development and analysis phase. The structure of the model, assumptions, input data, model settings and the type of analyses are described in detail in this section.

### Model overview

**Fig. 1:**
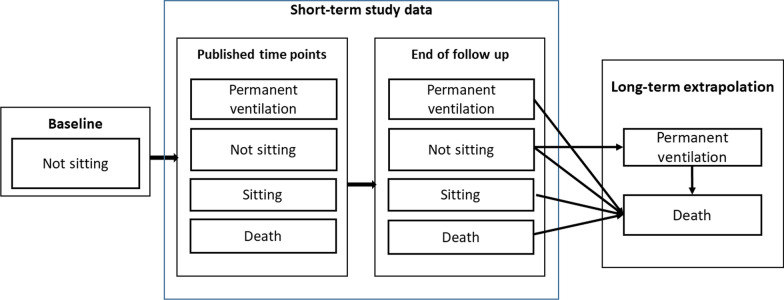
Model schematic

The health states in the model related to three constructs: the motor function milestones achieved, need for permanent ventilation, and death. The motor function milestones included sitting and walking. Other motor function milestones such as head control, rolling, crawling, and standing were not modelled as explicit health states, but health benefits associated with such improvements were explored as described in “Health State Utilities Section”. Figure 1 depicts the analytic framework for the model.

The model used monthly time cycles to estimate lifetime costs, life years (LYs) and quality adjusted life years (QALYs). The model contained two parts: (1) a short-term phase concordant with clinical study data, and (2) a long-term extrapolation model. A brief description of each is provided here, with detailed explanations on assumptions and data presented in subsequent sections.

Short-term data inputs for nusinersen and BSC were derived from the ENDEAR trial and SHINE study. [15, 22] These data were used directly in the model to capture the proportion of the patients in the different health states at different points in time, to allow estimation of the costs, LYs, and QALYs for the two strategies within the study periods.

The long-term model involved the extrapolation of motor function milestones, permanent ventilation, and mortality, the latter of which was assumed to be conditional on health states. In the base-case analysis, the motor function milestones achieved at the end of follow-up in the clinical trials were assumed to be sustained until death (i.e., patients stayed in the same motor function milestone-based health state until death). In addition, alternative scenarios were also modeled for nusinersen, where a proportion of patients lost milestones.

Transition to the “permanent ventilation” health state in the model was only possible for patients who did not have any motor function milestones (i.e., those in the “not sitting” health state). For these patients, both overall survival (OS) and ventilation-free survival (VFS) were modelled. Patients who achieved motor function milestones were not considered to be at risk of transitioning to “permanent ventilation”. As such, only OS was modelled for the patients with motor function milestones.

#### Key assumptions

Several key assumptions were made during the modelling phase, as listed below. A comprehensive list of assumptions and accompanying rationales is available in Additional file 1. Appendix S1.

Data from the trials and studies were used directly in the short-term model. Motor-function milestones achieved at the end of follow up were sustained until death. Only patients in the “not sitting” health state could transition to the “permanent ventilation” state. In the short-term model for nusinersen, it was assumed that the observed proportions of patients who could sit and attend follow-up visits was generalisable to all patients alive.

In the BSC arm, a partitioned survival modeling approach was used at the end of the short-term model to estimate transitions to “death” and “permanent ventilation” from the “not sitting” health state. In the nusinersen arm, we assumed that patients in the “not sitting” health state at the end of the short-term model had the same survival as those on “permanent ventilation”. This assumption may be favorable to nusinersen given that observational data suggest lower mortality for patients on permanent ventilation compared to those who were unable to sit.

In the clinical trials, patients on nusinersen achieved interim milestones such as head control, rolling, crawling, etc. Given these interim milestones were not explicitly captured in our model, additional utility benefits were assumed in the nusinersen arm. An additional utility benefit of 0.05 and 0.1 was attributed to the patients in the “not sitting” and “sitting” health states in the nusinersen arm, respectively.

A treatment-stopping rule at 24 months was assumed for patients on nusinersen who were in “not sitting” and “permanent ventilation” health states.

### Model inputs

The model inputs for the short-term data, long-term extrapolation, health state utilities, costs, and productivity gains are presented in the next subsections.

#### Short-term model

##### Motor function milestones

The data on proportions of nusinersen patients achieving motor function milestones at different time points for the different strategies were based on the ENDEAR trial [9] and the OLE SHINE study. [12] Castro et al. [12] reported the results of the SHINE study which presents the proportion of patients achieving sitting at different time points, as shown in Table 1.

**Table 1:** Motor function milestones achieved on nusinersen

	Baseline n = 81	Day 64 n = 70	Day 183 n = 65	Day 302 n = 51	Day 394 n = 48	Day 578 n = 31	Day 698 n = 17
% Achieving independent sitting (but not walking)	0	1	5	10	15	29	24
% Achieving walking	0	0	0	0	0	0	0

With different numbers of patients at risk at these time points, we followed a multi-stage process to estimate the true proportions of nusinersen patients achieving the milestones as described in Additional file 1. Appendix S2.

No patients in the BSC arm were assumed to achieve any motor function milestones at any time points, as the trial reported that 0% of the patients in the sham control group achieved the ability to sit independently during assessments at days 183, 302, or 394. We could not include longer-term data on this estimate in the BSC arm as all sham control patients in ENDEAR [9] switched to nusinersen treatment in SHINE. [12].

##### Mortality

The proportions of patients alive at different time points were estimated from the OS data presented for each strategy. The OS data for nusinersen were from patients who received nusinersen in both ENDEAR [9] and SHINE. [12] The OS data for BSC were from patients who received sham control in ENDEAR.

##### Permanent ventilation

The VFS rates at different time points were estimated from the combined VFS data in ENDEAR [9] and SHINE, [12] and subtracted from the OS data to estimate the proportion of patients receiving permanent ventilation for the nusinersen arm. The VFS data for BSC were from patients who received sham control in ENDEAR [9] alone.

##### Not sitting

In the short-term model, the proportion of patients in the “not sitting” health state was estimated as the complement of the sum of proportions of patients on permanent ventilation, patients achieving milestones, and patients who died.

#### Long-term model

##### Extrapolation of motor function milestones

Motor function milestones in the long-term model were extrapolated based on milestone status at the end of the short-term model, with a base-case assumption that milestone status remained the same until death.

Alternative scenarios were also modeled where it was assumed that a proportion (ranging from 10 to 30%) of patients in the “sitting” health state lost their motor function milestones.

##### Extrapolation of mortality and permanent ventilation

At the end of the short-term model, patients were in one of the following health states: “permanent ventilation,” “not sitting,” “sitting,” or “walking.”

Those in the “not sitting” health state in the BSC arm could transition either to “permanent ventilation” or “death” health states, and we modeled both OS and VFS for these patients. For those in the “not sitting” health state in the nusinersen arm, we modeled transition to only “death” (i.e., not to permanent ventilation). However, we included the costs of permanent ventilation in the 3 months prior to death for those transitioning to death from this health state.

The patients in all other health states were not considered to be at risk of transitioning to “permanent ventilation” and, as such, could only transition to “death”.

The long-term risks of mortality associated with each of the health states were modelled by fitting survival curves to digitized, published Kaplan–Meier (KM) data most relevant to each health state. We digitized the KM data and reconstructed the individual data using the methods described in Guyot et al. [17] We fitted different parametric distributions (exponential, Weibull, gamma, Gompertz, log-normal, log-logistic, and generalized gamma) to these survival data. We identified the best fitting curves based on a combination of clinical plausibility, fit statistics (Akaike information criteria (AIC) and Bayesian information criteria (BIC)), and visual inspection. For each health state, a single parametric distribution was selected to calculate the estimated probability of death in each cycle.

**Fig. 2:**
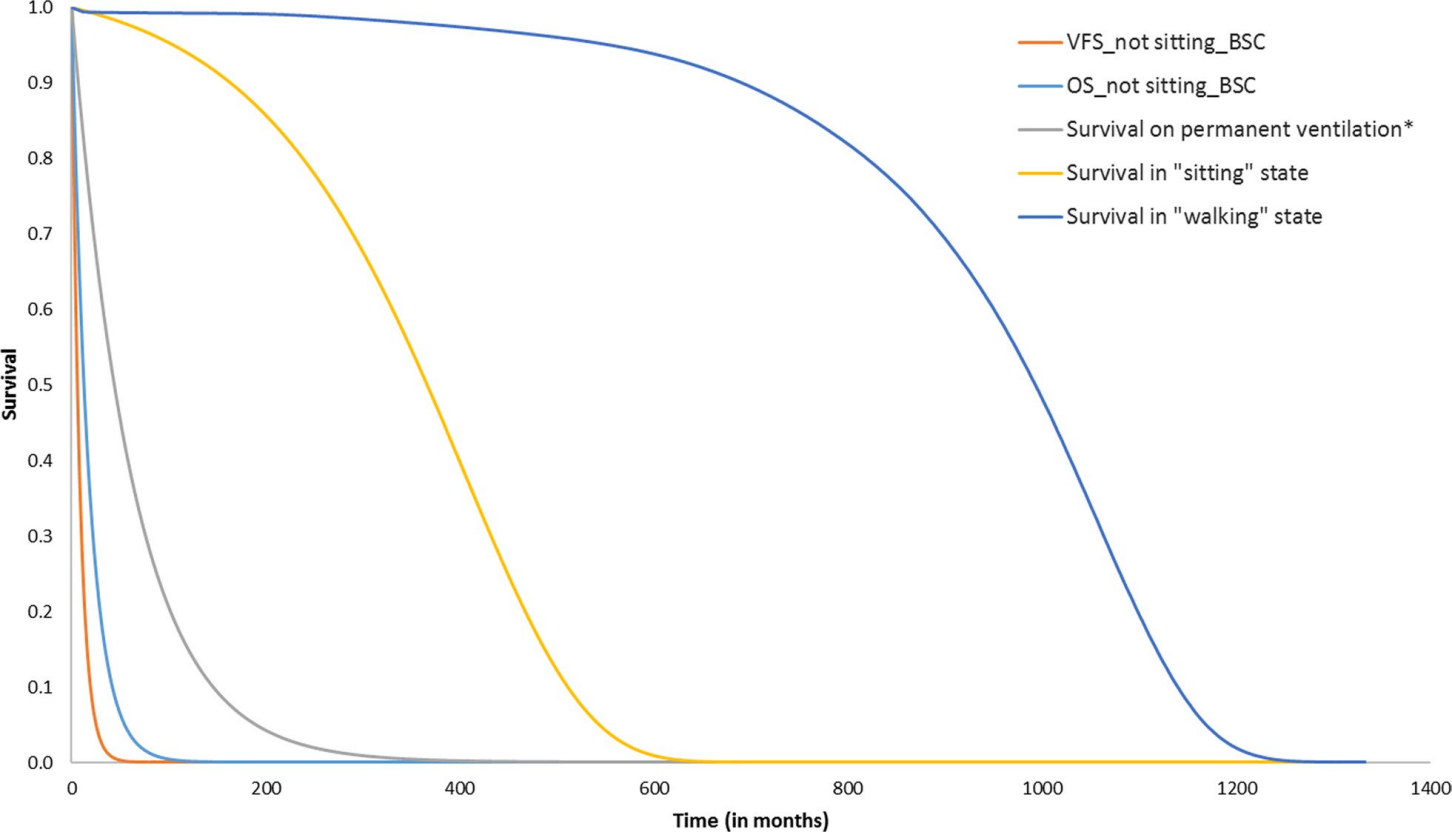
Survival curves used in the long-term extrapolation model. *BSC* best supportive care, *OS* overall survival, *VFS* ventilation-free survival.*Survival in “not sitting” health state in treatment arm is the same as survival on permanent ventilation

The survival curves used in the base-case analysis for long-term extrapolation are presented in Fig. 2. The transitions from different health states, assumptions, data sources, and parametric distributions selected to extrapolate survival are presented in Additional file 1. Appendix S3.

In Fig. 2, the OS and VFS curves represent the overall survival and ventilation-free survival of the patients in the “not sitting” health state in the BSC arm, which were assumed to be the same as that of the patients in the sham control arm of ENDEAR. The OS curve for BSC represents the survival of patients in the “not sitting” health state at the end of the short-term model, with a mean survival time of 1.55 years. The VFS curve, with a mean survival of 0.74 years, is subtracted from the OS curve to estimate the proportion of patients in the “permanent ventilation” health state that transitioned from the “not sitting” health state in each cycle.

The curve “survival on permanent ventilation” represents the survival of patients in the “permanent ventilation” health state at the end of the short-term model, with a mean survival of 5.3 years. The survival in the “not sitting” health state in the nusinersen arm was assumed to be the same as the survival on “permanent ventilation”, to account for the survival benefit in the treatment arms for achieving interim milestones such as head control and rolling among patients in the “not sitting” health state.

The curve “survival in sitting state” represents the survival of patients in the “sitting” health state at the end of the short-term model, based on the assumption that they have the same survival as SMA Type II patients, with a mean survival of 29.3 years.

#### Health state utilities

##### Patient utilities

The utilities used in the base-case analyses were derived from multiple sources and are presented in Table 2. The utilities reported by Thomson et al [18] were from a cross-sectional study of individuals with SMA in Europe; investigators collected parent/proxy–assessed quality of life using the EuroQol-5 Dimensions (EQ-5D) 3-level version. The mean utility value for patients with Type I SMA in the UK was 0.19 (n = 7); we assumed this value was the same for both “permanent ventilation” and “not sitting” health states in the BSC arm.

**Table 2:** Patient utility values for health states

	Utility value (BSC arm)	Source	Utility value (nusinersen arm)	Source
Permanent ventilation	0.19	Thomson et al., 2017 [18]	0.19	Thomson et al., 2017 [18]
Not sitting	0.19		0.29	Assumption
Sitting	0.60	Tappenden et al., 2018 [19]	0.65	Assumption

The utility for the “sitting” health state was estimated as 0.60 from Tappenden et al., [19] in the evidence review group (ERG) report evaluating the submission of nusinersen for National Institute for Health and Care Excellence (NICE). Tappenden et al. [19] report the utilities elicited from the clinical experts who advised the ERG, who were asked to provide plausible utility estimates for the different health states; it should be noted that these utility estimates were not preference-based.

Additional utility benefits in the nusinersen arm were assumed for achieving interim milestones such as head control, rolling, standing, crawling, etc. The proportions of patients achieving these interim milestones were not available at different time points, so the model assumed an additional utility benefit for all patients in the “not sitting” and “sitting” health states. This was implemented in the model as a utility of 0.29 for the “not sitting” health state (i.e., an additional utility of 0.10 compared with BSC) and a utility of 0.65 for the “sitting” health state (i.e., an additional utility of 0.05 compared with BSC) in the nusinersen arm.

#### Cost inputs

The costs used in the model include treatment costs, administration/monitoring costs, and costs associated with being in each health state. All costs were inflated to 2017 values.

##### Drug acquisition costs

The recommended dosage for nusinersen is four loading doses (the first three loading doses administered at 14 day intervals with the fourth loading dose administered 30 days after the third dose) and a maintenance dose administered once every 4 months thereafter. Since nusinersen is administered in a hospital setting, mark-ups associated with the treatment were included. Average wholesale price (AWP) was used to which a 15% discount was applied, reflecting the weighted average mark-ups seen for treatments administered specifically in a hospital outpatient setting. [21].

##### Administration and monitoring costs

All administration, laboratory, and monitoring costs associated with nusinersen are presented in Table 3. It was assumed that 40% of the patients receive nusinersen in an inpatient setting and accrue the costs of inpatient stay and anesthesia. More details about these costs are presented in Additional file 1. Appendix S4.

**Table 3:** Treatment and administration cost inputs

Strategy	Administration	Package size	WAC* per package	Estimated net cost per package^†^	Source
Nusinersen treatment cost	Intrathecal injection	2.4 mg/ml (5 ml)	$125,000	$127,500	Magellan 2016 [20]; Redbook 2018 [21]
Administration cost	$1209	Assuming 40% of patients receive nusinersen in inpatient settings			Physician fee schedule 2018; [22] Nationwide Children’s Hospital [23]

*Wholesale acquisition cost (WAC) as of July 1, 2019

^†^AWP–15%, where AWP is $150,000 per package as of July 1, 2019

##### Health state costs

The monthly costs associated with the different health states are presented in Table 4. The health care sector perspective included just the health care utilization costs while the non-medical costs were also included in the modified societal perspective.

**Table 4:** Monthly Costs in Different Health States

	Permanent ventilation	Not sitting	Sitting	Walking
Health care utilization costs	$28,218	$25,517	$6357	$2499
Non-medical costs*	$964	$964	$964	$0

*Used only in the modified societal perspective analyses

The health care utilization costs were sourced from claims analysis of commercial health plans reported by Shieh et al. [24] The costs in the “permanent ventilation” health state were estimated as the costs associated with permanent ventilation added to the costs of the “not sitting” health state. More details of these health state costs are presented in Additional file 1. Appendix S4.

Annual non-medical costs associated with the different health states were obtained from a report by the Lewin Group, [25] and are summarized in Table 4. More details of the non-medical costs are presented in Additional file 1. Appendix S4.

##### Patient productivity gains

Patient productivity gains were included in a scenario analysis using the modified societal perspective. No productivity changes were assumed for those in the “permanent ventilation” and “not sitting” health states. For other health states, data from the Lewin Group report [25] on educational attainment for SMA patients were combined with data on income by education level in the US from the Bureau of Labor Statistics [26] to estimate the productivity gains as monthly income of $4450, as shown in Additional file 1. Appendix S4. These productivity gains were estimated from the age of 25 years until an age of 67 years which represents the age of retirement in the US.

### Model verification and validation

Model verification followed standard practices in the field. All mathematical functions in the model were tested to ensure they were consistent with the manuscript (and Additional file 1. Appendix materials). Test analyses with specific input values (e.g., all set to 0, or all set to 1, etc.) were conducted to ensure the model was producing findings consistent with expectations. Further, independent modelers tested the mathematical functions in the model as well as the specific inputs and corresponding outputs.

### Sensitivity and scenario analyses

One-way sensitivity analyses (OWSA) were performed using plausible ranges based on published data and expert opinion to identify the key drivers of model outcomes. Probabilistic sensitivity analysis (PSA) was performed by jointly varying all model parameters, using 1000 simulation runs. Due to the lack of data, the distributions used for costs and utilities in the PSA were mean values ± 20%. As such, the true uncertainty is likely to be different to that represented in our probabilistic analyses.

We also conducted scenario analyses using a modified societal perspective including non-medical costs, alternative utility estimates, alternative health state costs, alternative survival estimates, using a 10-year time horizon and using a lower (1.5%) discount rate. We also performed alternative scenario analyses not accounting for utility benefits of achieving interim milestones (such as head control, rolling, crawling, and standing) and another scenario where the patients lose milestones, and have lower survival and utility in the “sitting” health state.

## Results

The base-case results, results of the PSA, scenario analyses and OWSA are presented from the health care sector perspective.

### Base-case results

The breakdown of QALYs, LYs and costs according to health state for the different strategies are presented in Additional file 1. Appendix S5.

The total lifetime costs associated with nusinersen were approximately $3.9 million and were $790,000 for BSC (Table 5). Nusinersen produces greater QALYs and LYs (3.24 and 7.64, respectively) compared with BSC (0.46 QALYs and 2.40 LYs). This resulted in an incremental cost per QALY gained of approximately $1,112,000 and an incremental cost per LY gained of $590,000 for nusinersen compared with BSC.

**Table 5:** Base-Case Results for nusinersen versus BSC in the health care sector perspective

	Drug treatment costs^*^	Non-treatment health care costs^*^	Total costs^*^	QALYs	LYs	Incremental results	
						Cost/QALY gained^*^	Cost/LY gained^*^
Nusinersen	$2,231,000	$1,653,000	$3,884,000	3.24	7.64	$1,112,000	$590,000
BSC	$0	$789,000	$789,000	0.46	2.40	–	–

*BSC* best supportive care, *LY* life-year, *QALY* quality-adjusted life year

*Costs and cost-effectiveness ratios are rounded to the nearest $1000

### One-way sensitivity analyses results

**Fig. 3:**
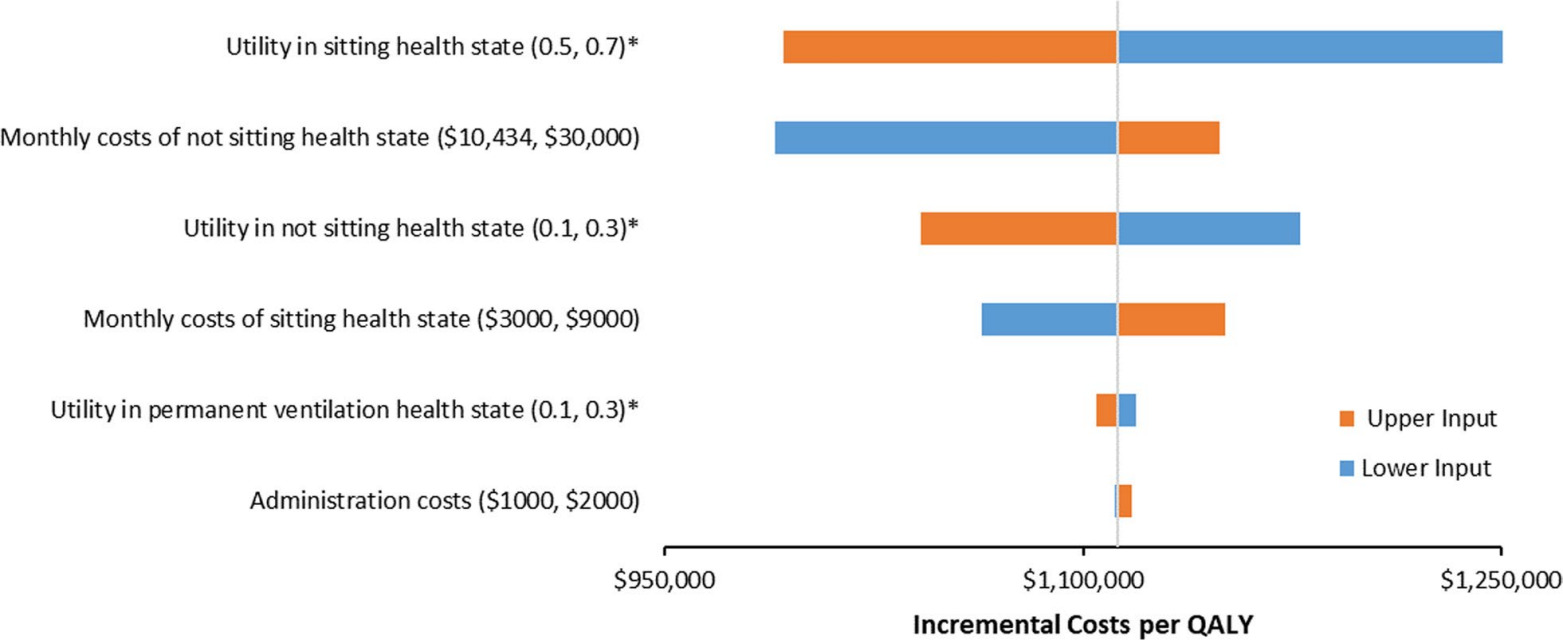
Tornado diagram for one-way sensitivity analyses of nusinersen versus BSC. *QALY* quality-adjusted life year. The values in the parenthesis in the y-axes represents the lower and upper input, respectively. *Lower input corresponds to higher incremental cost-effectiveness ratio and vice versa

The key drivers of uncertainty included monthly costs and utility values for the “sitting” and “not sitting” health states (Fig. 3).

### Probabilistic sensitivity analyses results

**Fig. 4:**
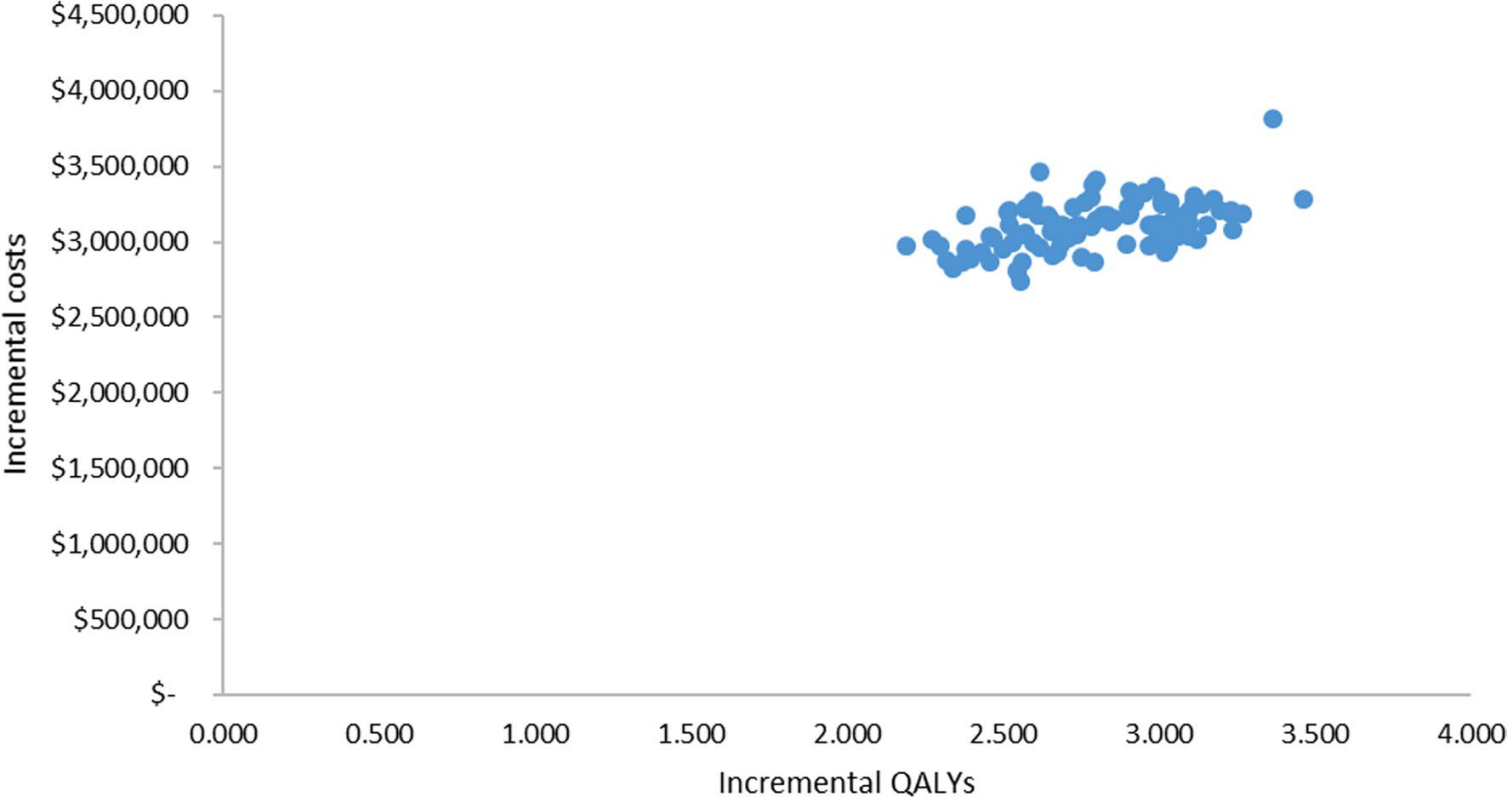
Cost-effectiveness clouds for nusinersen versus BSC

Figure 4 presents the cost-effectiveness clouds from the PSA for nusinersen versus BSC. The results of the PSA suggest that nusinersen had no likelihood of being cost-effective at thresholds less than $500,000 per QALY.

### Scenario analyses results

A number of scenario analyses were performed to identify the effect of alternative inputs and assumptions on the cost-effectiveness results.

Table 6 presents the results from a scenario analysis taking a modified societal perspective. The incremental cost per QALY and incremental cost per LY gained for nusinersen compared to BSC in the modified societal perspective were slightly less favorable than those in the health care sector perspective. This was because non-medical costs (which included moving or modifying the home and purchasing or modifying a vehicle), provided in Table 4, accrue for all the health states (except walking) for a lifetime, while patient productivity gains are only for patients sitting or walking between ages 25 and 67 years. The productivity gains did not offset the non-medical costs for nusinersen, as only around 19% of the patients in nusinersen arm were in the “sitting” health state and none were in the “walking” health state.

**Table 6:** Scenario analysis results for nusinersen versus BSC: modified societal perspective

	Total costs*	QALYs	LYs	Incremental results	
				Cost/QALY gained*	Cost/LY gained*
Nusinersen	$3,944,000	3.24	7.64	$1,124,000	$596,000
BSC	$817,000	0.46	2.40	–	–

*BSC* best supportive care, *LY* life-year, *QALY* quality-adjusted life year

*Costs and cost-effectiveness ratios are rounded to the nearest $1000

The summary results for the other scenario analyses conducted are presented in Table 7. More detailed description of the assumptions behind each of these scenario analyses and detailed results are in Additional file 1. Appendix S6.

**Table 7:** Scenario Analyses for nusinersen versus BSC

	Cost per QALY*	Cost per LY*
Base-case results	$1,112,000	$590,000
Scenario #1: Assuming no utility benefits for interim milestones	$1,303,000	$590,000
Scenario #2: Assuming lower monthly health state costs of $10,434 and $13,135 for “not sitting” and “permanent ventilation” health states, respectively	$990,000	$525,000
Scenario #3: Assuming lower utility of 0.5 for “sitting” health state	$1,265,000	$590,000
Scenario #4: Assuming lower survival (mean survival of 15.6 years) for “sitting” health state	$1,253,000	$624,000
Scenario #5: Assuming lower utility of 0.5 and lower survival (mean 15.6 years) for “sitting” health state	$1,407,000	$624,000
Scenario #6a: Assuming 10% in “sitting” health state lose milestone at end of short-term model	$1,143,000	$593,000
Scenario #6b: Assuming 20% in “Sitting” Health State Lose Milestone at End of Short-Term Model	$1,178,000	$597,000
Scenario #6c: Assuming 30% in “sitting” health state lose milestone at end of short-term model	$1,218,000	$601,000
Scenario 7: Scenario assuming 30% in “sitting” health state lose milestone at end of short-term model, lower utilities and survival for “sitting” health state	$1,509,000	$630,000
Scenario #8: Using a 10 year time horizon	$1,460,000	$700,000
Scenario #9: Using 1.5% discount rate for both costs and QALYs	$1,052,000	$566,000

*LY* life-year, *QALY* quality-adjusted life year

*Costs and cost-effectiveness ratios are rounded to the nearest $1000

Removing utility benefit for achieving interim milestones increased the incremental cost per QALY. Assuming lower health state costs resulted in more favorable incremental cost per QALY ratios. However, assuming lower survival or utilities for “sitting” health states resulted in less favorable incremental cost-effectiveness ratios. When both poorer survival and lower utilities for the “sitting” health state were used, the incremental cost per QALY gained was around $1.4 million. This suggests that the base-case incremental cost per QALY is an underestimate if the patients achieving “sitting” do not do as well as SMA Type II patients.

If a larger proportion of patients in the “sitting” health state were to lose their milestones, the incremental cost-effectiveness ratios become less favorable (scenarios #6a-6c in Table 7). The scenario which assumed that 30% of the patients in the “sitting” health state lose milestones and also assumed lower survival and lower utilities for those in the “sitting” health state, resulted in an incremental cost per QALY of approximately $1.5 million and an incremental cost per LY gained of $630,000. Note that this scenario still includes the utility benefit for achieving interim milestones.

The scenario analyses using a 10 year time horizon resulted in an incremental cost per QALY of approximately $1.5 million as all the benefits for the patients in the “sitting” health state are not included. The scenario analyses using a discount rate of 1.5% for both costs and QALYs resulted in an in incremental cost per QALY of approximately $1 million.

## Discussion

### Summary

This study represents the first de novo cost-effectiveness model of infantile-onset SMA patients in the US setting. The base-case incremental cost-effectiveness results were approximately $1.1 million per QALY and $600,000 per LY compared with BSC.

The incremental cost effectiveness ratios did not fall below $990,000 per QALY gained (or $520,000 per LY gained) in any of the analyses undertaken. The results were most sensitive to the length of survival, the costs associated with treating people with SMA, and the utilities in both the “sitting” and “not sitting” health states. Results from the probabilistic sensitivity analyses found that nusinersen had a zero likelihood of achieving a cost-effective ratio of less than $500,000 per QALY gained.

### Comparison to other models

A recently published manufacturer-funded model compared nusinersen to best supportive care in early-onset (Type I) SMA patients in Sweden. The cost-utility model was developed from a societal perspective, with a health care perspective analysis undertaken as a scenario, using a 40 year time horizon. [27] That model structure was also similar to the manufacturer-submitted models to NICE, CADTH, and other HTA agencies, with changes mainly to the patient utilities used and costs to match the respective jurisdiction. In our model, the incremental cost-effectiveness ratio was approximately $1.1 million per QALY while the corresponding results were substantially more favorable in the manufacturer-funded model, at approximately SEK 5.6 million ($623,000) per QALY.

This difference is primarily due to the more favorable assumption in the manufacturer-funded model of potentially continuous improvement with nusinersen beyond the trial duration. Both models employed health states based on motor function milestones, but beyond the trial period, our model assumed patients remained in the same health state as at end of trial, whereas in the manufacturer-funded model, it was assumed that patients receiving nusinersen could only improve or remain stable in each cycle, while patients in the BSC arm could not improve over time but could only worsen or stay within the same health state. This assumption of continuous improvement with nusinersen beyond the trial duration in the manufacturer-funded model was also questioned by the independent ERG and noted by the appraisal committee in the NICE appraisal. [28].

### Limitations

Our analyses have important limitations. Most of these relate to the lack of availability of robust data and the assumptions required to overcome this. There is no long-term follow-up, resulting in considerable uncertainty related to the prognosis of patients with SMA. We used motor function milestones to define broad health states and had to assume relationships between these motor function milestone-based health states and survival. Uncertainty in long-term survival was partially accounted for in sensitivity and scenario analyses. As there are no long-term data on the extrapolation of motor function milestones, the base-case analyses assume that these are sustained until death. Given nusinersen is a lifelong treatment it is possible that some patients may achieve further milestones in the longer term. On the other hand, it is possible that the patients may lose the milestones achieved. As such, in the absence of long-term follow-up data for nusinersen, the base-case analyses assume that these are sustained until death. However, as reported in Table 7, we performed scenario analyses assuming a proportion of the patients in the “sitting” health state lose their milestones to account for the possibility of deteriorating treatment effect over time. Given the lack of long-term follow up of treatment effectiveness and utility data, a registry of SMA patients is recommended.

Furthermore, some relevant interim motor function milestones (such as head control, crawling, rolling) were not included in the model. Given patients on nusinersen also achieved interim milestones, the base-case analyses included a utility benefit for patients receiving nusinersen compared to those receiving BSC to make allowances for better functioning in nusinersen arm within these broad health states. However, we also performed scenario analysis excluding this utility benefit associated with the interim milestones (please see Scenario 1 in Table 7).

We could not estimate disease progression parameters (e.g., transition probabilities) without access to individual patient data from the studies. As such, the data for the different strategies during the study period were used directly in the model to estimate short-term costs and QALYs. This is subject to limitations, especially towards the end of the follow up period, where censoring has reduced the relatively small numbers recruited in the studies.

Robust utility data were lacking, with many identified studies lacking face validity. As such, we used utility data derived from several sources that were believed to be coherent. The base-case analyses were complemented with sensitivity and scenario analyses to explore the uncertainty in these values. Similarly, cost data were lacking, requiring several assumptions to be made. These uncertainties were partially addressed through altering the cost inputs in sensitivity analyses, as well as presenting threshold-based price ranges. However, due to the lack of data, the distributions used for costs and utilities in the PSA are mean values ± 20%. As such, the true uncertainty is likely to be different than that represented in our probabilistic analyses.

Given the nature of SMA, it is difficult to disentangle the adverse events due to treatment from the complications associated with SMA itself, which are already accounted for in the health state costs and disutilities. As such, the costs and disutilities of adverse events were not included in the model.

Finally, our analyses using a modified societal perspective did not include quality of life burden associated with caregivers, as the methods for performing economic evaluations including such caregiver burden are still under development. Incorporating caregiver burden may lead to counterintuitive results due to prolonged negative productivity effects and unknown quality of life effects on caregivers when children who need substantial care live longer. Furthermore, there is a lack of data on utilities and lost income for caregivers of patients with SMA. As such, we did not include caregiver burden in the analyses using a modified societal perspective.

Onasemnogene abeparvovec-xioi (Zolgensma) is another potential treatment option for the infantile-onset SMA patients. [29] The evidence for Zolgensma in infantile-onset SMA is based on a single-armed study recruiting 12 patients, and there are no head to head trials comparing Zolgensma with nusinersen. There are also differences in study populations related to age at treatment initiation and disease duration that limit our ability to adequately distinguish the net health benefit, and consequently cost-effectiveness, of Zolgensma versus nusinersen for infantile-onset SMA. Given these considerations, we did not feel it was appropriate to include a comparison of Zolgensma versus nusinersen in our analysis and have focused our manuscript on nusinersen in infantile-onset SMA patients.

## Conclusions

This study represents the first de novo cost-effectiveness model of infantile-onset SMA patients in the US. In our base-case analysis, nusinersen produces greater QALYs and LYs (3.24 and 7.64, respectively) compared with BSC (0.46 QALYs and 2.40 LYs), resulting in an incremental cost per QALY gained of approximately $1,112,000 and an incremental cost per LY gained of $590,000 for nusinersen compared with BSC. Cost-effectiveness was dependent on the assumptions made about survival, costs, and utilities, and whether the motor function milestones were sustained over lifetime. At its current price, nusinersen does not meet traditional cost-effectiveness thresholds in the US. Given the relatively short-term effectiveness and utility data available, a registry to collect long-term data relating to efficacy and utility within infantile-onset SMA patients on treatment is recommended to allow a more accurate estimate of cost-effectiveness.

## Supplementary information

**Additional file 1:** **Appendix S1.** Key model choices and assumptions. **Appendix S2.** Estimating proportions of “sitting” patients on nusinersen. **Appendix S3.** Long term extrapolation. **Appendix S4**. Costs. **Appendix S5.** Breakdown of the results. **Appendix S6.** Scenario analyses results.

## Data Availability

Not applicable.
