# Supplementary material for: Cost effectiveness of nusinersen for patients with infantile-onset spinal muscular atrophy in US
Source: Cost Eff Resour Alloc. 2020 Oct 6;18:41. doi: 10.1186/s12962-020-00234-8 (PMC7539471; doi:10.1186/s12962-020-00234-8)
Supplement: Supplementary file 1 — Additional file 1: Appendix S1. Key model choices and assumptions. Appendix S2. Estimating proportions of “sitting” patients on nusinersen. Appendix S3. Long term extrapolation. Appendix S4. Costs. Appendix S5. Breakdown of the results. Appendix S6. Scenario analyses results. [file 12962_2020_234_MOESM1_ESM.docx]

### Appendix 1: Key Model Choices and Assumptions

##### Table A1. Key Model Assumptions and Rationale

| **Assumption** | **Rationale** |
| --- | --- |
| Data from the trials and studies on motor function milestones, permanent ventilation, and mortality were used directly in the short-term model. | Robust estimation of disease progression parameters (e.g., transition probabilities) was not possible without access to IPD from the trials and studies. As such, data for the different interventions during the study period were used directly in the model to estimate short-term costs/QALYs. |
| In the short-term model for nusinersen, it was assumed that the proportion of patients sitting among those alive who are not followed up is the same as the observed proportion of patients sitting among who attended the follow up visits. | The proportion of patients reported sitting in Castro et al*.*^1^ are based on those attending the follow-up visits at that time point and the proportion of patients who were able to sit among those who did not attend follow up visits is not known. As such, it was assumed that they are same. |
| Motor function milestones achieved at the end of the follow up are sustained until death. | There were no long-term data on the extrapolation of motor function milestones identified; the base-case analyses assume that these milestones are sustained until death. However, alternative scenario analyses were also considered. |
| Utility benefit was assumed in the treatment arms for patients achieving interim motor function milestones such as head control, rolling, crawling, and standing. | Although interim milestones are not modelled as explicit health states in the model, utility benefit was assumed in the treatment arms to account for achieving these interim milestones. This was implemented as additional utility benefit in treatment arms for the “not sitting” and ”sitting” health states. |
| Only patients in the “not sitting” health state can transition to “permanent ventilation” state. | Clinical experts deemed it reasonable to assume that patients achieving motor function milestones are not at risk of permanent ventilation. |
| In the BSC arm, for patients in the “not sitting” health state at the end of the short-term model, a partitioned survival modelling approach was used to estimate the proportions of patients dying and moving to permanent ventilation. | The data sources only reported the OS and the VFS, so the VFS curve is subtracted from the OS curve to estimate the proportion of patients in “permanent ventilation” health *s*tate. |
| In the treatment arms, it was assumed that patients in the “not sitting” health state at the end of the short-term model had the same survival as those on “permanent ventilation”. | The data show better survival in “permanent ventilation” state than “not sitting” state. As such, an assumption was made to account for the survival benefit in the treatment arms for achieving interim milestones such as head control and rolling among patients in the “not sitting” health state. This is an assumption favorable to the drug given that observational data suggest lower mortality for patients on permanent ventilation compared to those who were unable to sit. |
| No explicit transitions from “not sitting” to “permanent ventilation'” were modelled in the treatment arms. | The transition between these two health states is not known. However, additional costs for permanent ventilation were included for three months prior to death in the “not sitting” state |
| Patients with SMA Type I who are in “sitting” health state are assumed to have mortality similar to that of SMA Type II patients. | Clinical experts deemed it reasonable to assume that SMA Type I patients who can sit have similar prognosis as SMA Type II patients who are able to sit but not walk. |
| Patients with SMA Type I who are in “walking” health state are assumed to have mortality similar to that of SMA Type III patients. | Clinical experts deemed it reasonable to assume that SMA Type I patients who can walk have similar prognosis to SMA Type III patients who are able to walk. |
| Patients on nusinersen who did not achieve motor function milestones at 24 months discontinued the treatment. No other patients were assumed to discontinue nusinersen in the model. | In the nusinersen model submitted to the National Institute for Health and Care Excellence (NICE), this was assumed to be 13 months. However, our model used 24 months to reflect the patients who continue to receive nusinersen, as observed in SHINE^1^ extension study. |
| AE costs and disutilities were not included in the model. | Given the nature of SMA, it is difficult to disentangle the AEs due to treatment from the complications associated with SMA, which are already accounted for in the health state costs and disutilities. As such, separate costs and disutilities for adverse events are not included in the model. |
| The costs of BSC are not broken out beyond the health state costs in the model. | It is likely that the health state costs included in the model already include the costs of BSC. |
| The transition probabilities were not adjusted for age at the start of treatment in the SMA Type I model. | The data sources used to estimate the mortality risks for SMA Type I patients have similar starting ages, so they are not explicitly adjusted for age at treatment. |

### Appendix 2: Estimating Proportions of “Sitting” Patients on Nusinersen

Whilst we know the proportion sitting among those who attended the follow up visits, we do not know the proportion of patients sitting among those who did not. Given this, we assumed that the patients who did not attend the follow up have a similar proportion sitting as those who attended the follow up visits. Note that this is an assumption favorable to nusinersen as in reality it is likely that those who are in permanent ventilation have less likelihood to move to sitting health state compared to those who are in not sitting health state. As such, we multiplied the proportions of nusinersen patients alive at each of the time points and with the proportions of patients sitting at each time point to estimate the proportion sitting in nusinersen at different time points.

For estimating the proportion of nusinersen patients alive over time, we digitized the KM curve for OS in SHINE to estimate the survival at different time points. The manufacturer (Biogen) also provided us, as academic in confidence, data on number of patients deceased at each of the follow up visits. We used the data given by Biogen to estimate the proportion alive at the follow up visit time points and for the other time points we used the approximated survival estimates from the digitized KM curve.

As the data on proportion sitting in Castro et al poster is presented as integers, we followed a multi-stage process to estimate the true proportions of nusinersen patients sitting at the different time points. In step one, the numbers of patients sitting at each time point were estimated. In step two, these were rounded to the nearest integer. In step three, these integer values representing the number of patients sitting were divided by the number of patients at risk at each time point to estimate the true proportions of patients sitting. Also, to match with the model structure, the days at the follow up visits were converted into months and rounded to the nearest integer.

##### Table A2. Estimating Proportions of “Sitting” Patients at Different Time Points on Nusinersen

|  | Baseline  Month 0  n=81 | Day 64  Month 2  n=70 | Day 183  Month 6  n=65 | Day 302 Month 10  n=51 | Day 394 Month 13  n=48 | Day 578 Month 19  n=31 | Day 698  Month 23  n=17 |
| --- | --- | --- | --- | --- | --- | --- | --- |
| % Achieving Independent Sitting (But Not Walking) | 0 | 1 | 5 | 10 | 15 | 29 | 24 |
| Step 1: Estimating Numbers of Patients at Each Period | 0 | 0.7 | 3.25 | 5.1 | 7.2 | 8.99 | 4.08 |
| Step 2: Rounding the Numbers to the Nearest Integer | 0 | 1 | 3 | 5 | 7 | 9 | 4 |
| Step 3: Proportion Sitting in Those Attending Follow Up | 0.000 | 0.0143 | 0.0462 | 0.0980 | 0.1458 | 0.2903 | 0.2353 |
| % Sitting | 0.0000 | 0.0134 | 0.0399 | 0.0823 | 0.1206 | 0.2294 | 0.1859 |

### Appendix 3: Long term extrapolation

The model used health state-specific mortality risks for the proportion of patients alive at the end of the short-term model. The long-term risk of mortality associated with each of the health states was modelled by fitting survival curves to the digitized published Kaplan-Meier (KM) data most relevant to each health state. For each health state, a single parametric distribution was selected to calculate the estimated probability of death in each time period (i.e. each month).

The KM data was digitized, and the individual data were reconstructed using the methods described in Guyot et al.^2^ Different parametric distributions were fitted and the best fitting curves were identified based on a combination of: visual inspection, fit statistics such as Akaike information criteria (AIC)/Bayesian information criteria (BIC), and clinical plausibility. The mortality risks associated with each health state are described in detail below.

##### Table A3. Summary of the Long-Term Extrapolation

|  | **Description** | **Assumption** | **Source** | **Distribution Selected** | **Parameters** |
| --- | --- | --- | --- | --- | --- |
| **Not Sitting**  **(BSC Arm)** | OS | Assumed to be same as BSC patients | ENDEAR sham control arm^3^ | Exponential | λ_tw_=0.0127 |
|  | VFS | Assumed to be same as BSC patients | ENDEAR sham control arm^3^ | Exponential | λ_tw_=0.0276 |
| **Not Sitting**  **(Treatment Arms)** | OS | Assumed to be same as when on permanent ventilation | Gregoretti et al^4^ (NRA curve) | Exponential | λ_tm_=0.0158 |
|  | VFS | Not explicitly modelled | -- | -- | -- |
| **Permanent Ventilation** | Mortality | Assumed to be same as patients on non-invasive respiratory muscle aid, including non-invasive ventilation, tracheostomy, or mechanically assisted cough | Gregoretti et al^4^ (NRA curve) | Exponential | λ_tm_=0.0158 |
| **Sitting** | Mortality | Assumed to be same as SMA Type II patients | Zerres and Schöneborn et al.^5^ | Gompertz | α=0.0964, β=0.0037 |
| **Walking** | Mortality | Assumed to be same as general population | US population mortality^6^ | -- | -- |

#### Permanent Ventilation and Mortality from the “Not Sitting” Health State in the BSC arm

Patients from the “not sitting” state could transition to either the “permanent ventilation” health state or to death*.* At each monthly cycle, the ventilation free survival (VFS) curve was subtracted from the OS curve to estimate the proportion of patients in the “permanent ventilation” health *s*tate.

The source of data available to model these (i.e., VFS and OS) of SMA Type I patients was the sham control arm of the ENDEAR trial (n=41), with a follow-up of 52 weeks^3^. Exponential distributions were selected to model the VFS and OS based on clinical plausibility, visual fit, and AIC/BIC.

##### Table A4. Fit Statistics for Parametric Distributions Fitted to Overall Survival of Sham Control Arm in ENDEAR^3^

| Distribution | AIC | BIC |
| --- | --- | --- |
| Exponential | 185.79 | 187.50 |
| Weibull | 186.86 | 190.28 |
| Gompertz | 183.72 | 187.15 |
| Log-Normal | 183.87 | 187.29 |
| Log-Logistic | 185.42 | 188.85 |
| Gamma | 187.21 | 190.63 |
| Generalized Gamma | 180.00 | 185.14 |

AIC: Akaike Information Criteria, BIC: Bayesian Information Criteria

##### Figure A1. Parametric Distributions Fitted to Overall Survival of Sham Control Arm in ENDEAR^3^.

#### Not Sitting to Death or Permanent Ventilation

##### Table A5. Fit Statistics for Parametric Distributions Fitted to Ventilation Free Survival of Sham Control Arm in ENDEAR^3^.

| Distribution | AIC | BIC |
| --- | --- | --- |
| Exponential | 258.27 | 259.99 |
| Weibull | 260.11 | 263.54 |
| Gompertz | 259.48 | 262.91 |
| Log-Normal | 255.25 | 258.68 |
| Log-Logistic | 256.20 | 259.62 |
| Gamma | 259.69 | 263.12 |
| Generalized Gamma | 255.77 | 260.91 |

AIC: Akaike Information Criteria, BIC: Bayesian Information Criteria

##### Figure A2. Parametric Distributions Fitted to Sham Control Arm in ENDEAR^3^.

#### Mortality from the “Permanent Ventilation” Health State

We used retrospective data^4^ of SMA Type I patients from four Italian centers from 1992 to 2010 to model mortality in the “permanent ventilation” health state. In this study, 31 patients required continuous non-invasive respiratory muscle aid, including non-invasive ventilation and mechanically assisted cough (n=31). This data was used to model the mortality risk from the permanent ventilation state. Different parametric curves were fitted and exponential distribution was chosen based on visual inspection, fit statistics (AIC/BIC), and clinical plausibility.

##### Figure A3. Parametric Distributions Fitted to NRA Arm in Gregoretti et al.^4^

##### Table A6. Fit Statistics for Parametric Distributions Fitted to NRA Arm in Gregoretti et al.

| Distribution | AIC | BIC |
| --- | --- | --- |
| Exponential | 146.07 | 147.50 |
| Weibull | 147.78 | 150.65 |
| Gompertz | 148.00 | 150.87 |
| Log-Normal | 147.95 | 150.82 |
| Log-Logistic | 148.13 | 151.00 |
| Gamma | 147.79 | 150.65 |
| Generalized Gamma | 149.78 | 154.08 |

AIC: Akaike Information Criteria, BIC: Bayesian Information Criteria

#### Permanent Ventilation and Mortality from the “Not Sitting” Health State in the treatment arms

The patients in the “not sitting” health state in the treatment arms were assumed to have the same mortality as in the “permanent ventilation” health state. This is to account for the survival benefit of the “not sitting” patients in the treatment arms for achieving interim milestones such as head control and rolling. No explicit transitions from “not sitting” to “permanent ventilation” were modelled, however, additional costs for permanent ventilation were included for three months prior to death in the “not sitting” state.

#### Mortality from the “Sitting” Health State

Treated SMA Type I patients who can sit were assumed to have similar prognosis as SMA Type II patients who are able to sit but not walk. Pooled data from German and Polish studies on SMA Type II patients (n=240) presented in Zerres and Schöneborn et al.^5^ were used to model mortality from the “sitting” health state. We fitted to the early part of the KM curve as estimated by the algorithm in Guyot et al^2^ and assumed constant censoring over the entire time period. Different parametric curves were fitted and Gompertz distribution was chosen based on visual inspection, fit statistics (AIC/BIC), and clinical plausibility.

##### Figure A4. Parametric Distributions Fitted to Survival of SMA Type II Patients in Zerres and Schöneborn et al.^5^

##### Table A7. Fit Statistics for Parametric Distributions Fitted to Survival of SMA Type II Patients in Zerres and Schöneborn et al.^5^

| Distribution | AIC | BIC |
| --- | --- | --- |
| Exponential | 347.86 | 351.34 |
| Weibull | 327.96 | 334.92 |
| Gompertz | 335.64 | 342.60 |
| Log-normal | 325.92 | 332.88 |
| Log-logistic | 326.50 | 333.46 |
| Gamma | 326.53 | 333.49 |
| Generalized Gamma | 327.89 | 338.33 |

AIC: Akaike Information Criteria, BIC: Bayesian Information Criteria

#### Mortality from the “Walking” Health State

Treated patients with Type I SMA who can walk are assumed to have similar prognosis as patients with SMA Type III who are able to walk. A previously-conducted study^5^ reported no significant reduction in lifespan among SMA Type III patients compared to the general population. As such, we use the general population mortality^6^ for patients with Type I SMA who can walk.

### Appendix 4: Costs

#### Administration and Monitoring Costs

All administration, laboratory, and monitoring costs associated with the treatments are presented in Table A8. For nusinersen, it was assumed that 40% of the patients receive the treatment in an inpatient setting and accrue the costs of inpatient stay and anesthesia.

##### Table A8. Costs Associated with nusinersen Treatment

|  | **Cost** | **Description** | **Source** |
| --- | --- | --- | --- |
| Intrathecal Injection (Lumbar Puncture into Central Nervous System) | $82.44 | Current Procedural Terminology (CPT) code 96450 | Physician fee schedule 2018;^7^ facility price |
| Intrathecal Injection (Drain Cerebrospinal Fluid) | $86.76 | CPT 62272 |  |
| MD/Specialist | $52.20 | CPT 99213 |  |
| Monitor for Thrombocytopenia | $5.53 | CMS laboratory fee schedule 85049 |  |
| Monitor for Renal Toxicity | $10.72 | CMS laboratory fee schedule 80069 |  |
| Anesthesia for Lumbar Puncture | $133.13 | HCPCS 00635 |  |
| Imaging (Ultrasound or Fluoroscopy – Average Cost) | $78.66 | CPT 77003, 76942 |  |
| Inpatient Cost per Diem (Routine Surgery) | $1,316 | Using a cost:charge ratio of 1:3 | Nationwide Children’s Hospital^8^ |
| Inpatient Anesthesia | $583 | Using a cost:charge ratio of 1:3 |  |
| **Total Administration Cost** | $1,209 | Assuming 40% of patients receive nusinersen in inpatient settings | |

#### Health Care Utilization Costs

The monthly costs associated with the different health states are presented in Table A9. They were sourced from a claims analysis of commercial health plans comprising infantile-onset SMA (n=23), childhood-onset SMA (n=22) and later-onset SMA (n=296) patients, based on the study reported by Shieh et al.^9^ The costs of infantile SMA patients were used for the “not sitting” health state. The costs of childhood-onset SMA and later-onset SMA were used for the “sitting” and “walking” health states, respectively.

The costs in the “permanent ventilation” health state were estimated as the costs associated with permanent ventilation added to the costs of the “not sitting” health state. These included the costs of equipment and disposable equipment and supplies that are associated with ventilator-dependent children living at home, estimated from a UK study by Noyes et al.^10^ These costs were converted into US dollars using 2002 exchange rates^11^ and then inflated to 2017 dollars. The additional costs of permanent ventilation were estimated as $32,413 per year, which translates to an additional monthly cost of $2,701. In total, the monthly costs of the permanent ventilation health state were estimated as $28,218.

##### Table A9. Background Costs in Different Health States

|  | **Permanent Ventilation** | **Not Sitting** | **Sitting** | **Walking** |
| --- | --- | --- | --- | --- |
| Inpatient Hospitalization | $21,863 | $21,863 | $3,401 | $1,116 |
| Outpatient Services | $3,341 | $3,341 | $2,631 | $984 |
| Emergency Services | $313 | $313 | $325 | $399 |
| Costs Specific to Permanent Ventilation | $2,701 | -- | -- | -- |
| **Total Monthly Cost** | $28,218 | $25,517 | $6,357 | $2,499 |

Scenario analyses were performed using cost data from Armstrong et al.^12^ who reported additional total annual health care costs for patients with SMA diagnosed before and after one year of age, respectively. Scenario analyses were also performed using cost data from a report by the Lewin Group^13^ that reported additional total annual health care costs broken out for patients with early onset and other types of SMA.

#### Non-Medical Costs

Annual non-medical costs associated with the different health states were obtained from a report by the Lewin Group,^13^ and are summarized in Table A10. We excluded the “professional caregiving” costs from non-medical costs, as the costs in the “professional caregiving” category included some costs that we considered to medical (e.g., home health aides, skilled nurses, or nurse assistants) and others that may be incurred by health care payers (e.g., government programs, insurance, etc.). While this category also included some types of paid caregiving that would not be considered as medical (e.g., “relatives/friends who are paid by families or state programs to care for the affected persons”), the proportions of medical versus non-medical costs were not reported.

In a scenario analysis using a modified societal perspective, a weighted average of early onset and other SMA patients’ non-medical cost was used for all health states (except the walking health state, which had zero non-medical costs). The costs, which included moving or modifying the home and purchasing or modifying a vehicle, were estimated as mean annual costs but the follow-up period was not clear. Given this, these costs were assumed as recurring costs in the model, rather than stopping or changing over time.

##### Table A10. Monthly Non-Medical Costs

|  | **Permanent Ventilation** | **Not Sitting** | **Sitting** | **Walking** |
| --- | --- | --- | --- | --- |
| **Total Costs** | $964 | $964 | $964 | $0 |

#### Patient Productivity Gains

No productivity changes were assumed for those in the “permanent ventilation” and “not sitting” health states. For other health states, data from the Lewin Group report^13^ on educational attainment for SMA patients were combined with data on income by education level in the US from the Bureau of Labor Statistics^14^ to estimate the productivity gains of patients. These proportions were weighted by monthly earnings to estimate the potential monthly income as $4,450, as shown in Table A11 below. These productivity gains were estimated from the age of 30 years until an age of 65 years.

##### Table A11. Patient Productivity Gains

| Education Level | Numbers (n) (N=188) | Proportions (i.e., n/N) | Weekly Earnings* |
| --- | --- | --- | --- |
| Data Not Available | 8 | 0.0426 | $520† |
| Less than High School | 8 | 0.0426 | $520 |
| High School Graduate | 28 | 0.1489 | $712 |
| Some College/Associate Degree/Post-High School Education | 56 | 0.2979 | $836 |
| College Graduate | 51 | 0.2713 | $1,173^‡^ |
| Post-Graduate | 37 | 0.1968 | $1,660^§^ |
| Potential Monthly Income | $4,450 | | |

*https://www.bls.gov/emp/tables/unemployment-earnings-education.htm.

†Assumed to be the earnings of those who have less than high school diploma. ‡Assumed to be the earnings from bachelor’s degree.

§Assumed to be average of earnings from master’s degree, professional degree, and doctoral degree.

### Appendix 5: Breakdown of the Results

The breakdown of the LYs, QALYs, and costs according to health state for the different interventions in the SMA Type I population are presented here. Table A12 presents the breakdown for LYs. As can be observed, the majority of the LYs and QALYs gained are in the “sitting” and “walking” health states. This is because of the longer survival associated with these health states compared with the “not sitting” and “permanent ventilation” health states. None of the patients in BSC arm achieved milestones, and as such the LYs achieved in this arm were lower compared with the treatment arms. In the nusinersen arm, around 19% of the patients were in the sitting health state at the end of the short-term model, which provided 5.32 LYs.

##### Table A12. Undiscounted LYs by Health State in the SMA Type I Model

| Undiscounted LYs | Ventilated | Not Sitting | Sitting | Walking | Total Undiscounted LYs |
| --- | --- | --- | --- | --- | --- |
| BSC | 1.99 | 0.70 | 0.00 | 0.00 | 2.68 |
| nusinersen | 2.23 | 2.73 | 5.32 | 0.00 | 10.28 |

LY: life-year

The breakdown of the discounted LYs and QALYs according to health state for the different interventions are presented in Tables A13 and A14. These results follow the same pattern as Table A12, but the absolute numbers are lower due to discounting (for discounted LYs) and the use of QoL weights for discounted QALYs. The utility values in the “not sitting” and “permanent ventilation” health states were 0.19, resulting in quite low QALYs for BSC. For nusinersen, the majority of the QALYs are from the patients in the “sitting” health state, who have a utility of 0.6.

##### Table A13. Discounted LYs by Health State in the SMA Type I Model

| Discounted LYs | Ventilated | Not Sitting | Sitting | Walking | Total Discounted LYs |
| --- | --- | --- | --- | --- | --- |
| BSC | 1.71 | 0.68 | 0.00 | 0.00 | 2.40 |
| nusinersen | 1.89 | 2.40 | 3.36 | 0.00 | 7.64 |

LY: life-year

##### Table A14. Discounted QALYs by Health State in the SMA Type I Model

| Discounted QALYs Gained | Ventilated | Not Sitting | Sitting | Walking | Total Discounted QALYs |
| --- | --- | --- | --- | --- | --- |
| BSC | 0.33 | 0.13 | 0.00 | 0.00 | 0.46 |
| nusinersen | 0.36 | 0.70 | 2.18 | 0.00 | 3.24 |

QALY: quality adjusted life-year

The breakdown of the discounted costs according to health state for the different interventions are presented in Table A15. The costs are broken out into treatment costs, administration costs, and non-treatment health care costs.

For nusinersen, as seen in Table A15, treatment costs made up the majority of overall costs. Treatment costs were broadly proportional to the LYs gained in each health state; it should be noted that the model assumed that treatment is discontinued after 24 months for patients who do not achieve milestones (i.e., the patients in the “not sitting” and “permanent ventilation” states).

For BSC, health care costs were associated only with patients in the “not sitting” and “permanent ventilation” health states. The costs of permanent ventilation were higher for BSC, reflecting the longer survival of these patients.

Regarding the non-treatment health care costs, for nusinersen, most of the costs associated with the “sitting” health state were accrued in the short-term model, due to most patients starting in this state (while they achieve the milestones) and to these costs not being affected by discounting, as they are accrued at the beginning of the model. Again, the costs of permanent ventilation were higher for nusinersen, reflecting the longer survival of these patients.

##### Table A15. Breakdown of the Discounted Costs by Health State

| Treatment Costs | Ventilated | Not Sitting | Sitting | Walking | Total |
| --- | --- | --- | --- | --- | --- |
| BSC | -- | -- | -- | -- | -- |
| nusinersen | $156,569 | $794,619 | $1,279,642 | -- | $2,230,829 |
| Administration Costs | **Ventilated** | **Not Sitting** | **Sitting** | **Walking** | **Total** |
| BSC | -- | -- | -- | -- | -- |
| nusinersen | $1,485 | $7,535 | $12,134 | -- | $21,154 |
| Health Care Costs | **Ventilated** | **Not Sitting** | **Sitting** | **Walking** | **Total** |
| BSC | $580,684 | $208,793 | -- | -- | $789,477 |
| nusinersen | $641,516 | $733,869 | $256,173 | -- | $1,631,557 |
| *Placeholder price. | | | | | |

### Appendix 6: Scenario Analyses Results

We performed several scenario analyses to identify the effect of alternative inputs and assumptions on the cost effectiveness results. These are described in more detail below.

#### Scenario Analysis Assuming No Utility Benefits for Interim Milestones

Here, we assumed no utility benefits in the treatment arms for achieving interim milestones such as head control, rolling, standing, crawling, etc. This was implemented in the model as a utility of 0.19 for the “not sitting” health state and a utility of 0.65 for the “sitting” health state for both BSC and treatment arms. Table A16 presents the results for this scenario analysis. As expected, the QALY gains in the nusinersen arm are lower, resulting in higher incremental cost-effectiveness ratios compared to the base-case analyses.

##### Table A16. Results for Scenario Analysis Assuming No Utility Benefits for Interim Milestones

|  | Treatment Costs | Non-Treatment Health Care Costs | Total Costs | QALYs | LYs | Incremental Results | |
| --- | --- | --- | --- | --- | --- | --- | --- |
|  |  |  |  |  |  | **Cost/QALY Gained** | **Cost/LY Gained** |
| nusinersen | $2,231,000 | $1,653,000 | $3,884,000 | 2.83 | 7.64 | $1,303,000 | $590,000 |
| BSC | $0 | $789,000 | $789,000 | 0.46 | 2.40 | -- | -- |

BSC: best supportive care, LY: life-year, QALY: quality-adjusted life year

#### Scenario Analysis Assuming Lower Health State Costs for Not Sitting and Permanent Ventilation Health States

Table A17 present the results for the scenario analysis assuming lower costs of $10,434 for the “not sitting” health state and $13,135 for the “permanent ventilation” health state. As expected, the costs in the nusinersen arm are lower, resulting in lower incremental cost-effectiveness ratios compared to the base-case analyses.

##### Table A17. Results for Scenario Analysis Assuming Lower Health State Costs for Not Sitting and Permanent Ventilation

|  | Treatment Costs | Non-Treatment Health Care Costs | Total Costs | QALYs | LYs | Incremental Results | |
| --- | --- | --- | --- | --- | --- | --- | --- |
|  |  |  |  |  |  | **Cost/QALY Gained** | **Cost/LY Gained** |
| nusinersen | $2,231,000 | $877,000 | $3,108,000 | 3.24 | 7.64 | $990,000 | $525,000 |
| BSC | -- | $356,000 | $356,000 | 0.46 | 2.40 | -- | -- |

BSC: best supportive care, LY: life-year, QALY: quality-adjusted life year

#### Scenario Analysis Assuming Lower Utilities for Sitting Health State

Table A18 presents the results for the scenario analysis assuming lower utility of 0.5 for the “sitting” health state. As expected, the QALYs in the nusinersen arm are lower, resulting in higher incremental cost-effectiveness ratios compared to the base-case analyses.

##### Table A18. Results for Scenario Analysis Assuming Lower Utilities for Sitting Health State

|  | Treatment Costs | Non-Treatment Health Care Costs | Total Costs | QALYs | LYs | Incremental Results | |
| --- | --- | --- | --- | --- | --- | --- | --- |
|  |  |  |  |  |  | **Cost/QALY Gained** | **Cost/LY Gained** |
| nusinersen | $2,231,000 | $1,653,000 | $3,884,000 | 2.90 | 7.64 | $1,265,000 | $590,000 |
| BSC | -- | $789,000 | $789,000 | 0.46 | 2.40 | -- | -- |

BSC: best supportive care, LY: life-year, QALY: quality-adjusted life year

#### Scenario Analysis Assuming Lower Survival for Sitting Health State

Table A19 presents the results for the scenario analysis assuming roughly halved mean survival for the “sitting” health state, a mean survival of 15.6 years. This scenario was implemented using HR of 5 to the survival curve for “sitting” health state. As expected, the LYs and QALYs in the nusinersen arm are lower, resulting in higher incremental cost-effectiveness ratios compared to the base-case analyses.

##### Table A19. Results for Scenario Analysis Assuming Lower Survival for Sitting Health State

|  | Treatment Costs | Non-Treatment Health Care Costs | Total Costs | QALYs | LYs | Incremental Results | |
| --- | --- | --- | --- | --- | --- | --- | --- |
|  |  |  |  |  |  | **Cost/QALY Gained** | **Cost/LY Gained** |
| nusinersen | $1,807,000 | $1,564,000 | $3,371,000 | 2.52 | 6.53 | $1,253,000 | $624,000 |
| BSC | -- | $790,000 | $790,000 | 0.46 | 2.40 | -- | -- |

BSC: best supportive care, LY: life-year, QALY: quality-adjusted life year

#### Scenario Analysis Assuming Lower Survival and Lower Utility for Sitting Health State

Table A20 presents the results for the scenario analysis assuming roughly halved mean survival and lower utility for the “sitting” health state. As expected, the LYs and QALYs in the nusinersen arm are lower, resulting in higher incremental cost-effectiveness ratios compared to the base-case analyses.

##### Table A20. Results for Scenario Analysis Assuming Lower Survival and Utility in Sitting Health State

|  | Treatment Costs | Non-Treatment Health Care Costs | Total Costs | QALYs | LYs | Incremental Results | |
| --- | --- | --- | --- | --- | --- | --- | --- |
|  |  |  |  |  |  | **Cost/QALY Gained** | **Cost/LY Gained** |
| nusinersen | $1,807,000 | $1,564,000 | $3,371,000 | 2.29 | 6.53 | $1,407,000 | $624,000 |
| BSC | -- | $789,000 | $789,000 | 0.46 | 2.40 | -- | -- |

BSC: best supportive care, LY: life-year, QALY: quality-adjusted life year

#### Scenario Analysis Assuming Loss of Milestones in “Sitting” Health State

In this scenario, 10%-30% of the patients in the “sitting” health state of the nusinersen arm were assumed to drop a milestone. Tables A21-A23 present the results comparing nusinersen to BSC for these scenario analyses, respectively.

##### Table A21. Assuming 10% of Patients in the “Sitting” Health State Lose Milestone at the End of the Short-Term Model

|  | **Treatment Costs** | **Non-Treatment Health Care Costs** | **Total Costs** | **QALYs** | **LYs** | **Incremental Results** | |
| --- | --- | --- | --- | --- | --- | --- | --- |
|  |  |  |  |  |  | **Cost/QALY Gained** | **Cost/LY Gained** |
| **nusinersen** | $2,114,000 | $1,652,000 | $3,766,000 | 3.06 | 7.41 | $1,143,000 | $593,000 |
| **BSC** | -- | $789,000 | $789,000 | 0.46 | 2.40 | -- | -- |

In this scenario, 20% of the patients in the “sitting” health state of the nusinersen arm were assumed to drop in milestones. Table A22 presents the results for this scenario analysis.

##### Table A22. Assuming 20% of Patients in the “Sitting” Health State Lose Milestone at the End of the Short-Term Model

|  | **Treatment Costs** | **Non-Treatment Health Care Costs** | **Total Costs** | **QALYs** | **LYs** | **Incremental Results** | |
| --- | --- | --- | --- | --- | --- | --- | --- |
|  |  |  |  |  |  | **Cost/QALY Gained** | **Cost/LY Gained** |
| **nusinersen** | $1,996,000 | $1,652,000 | $3,648,000 | 2.88 | 7.18 | $1,178,000 | $597,000 |
| **BSC** | -- | $789,000 | $789,000 | 0.46 | 2.40 | -- | -- |

In this scenario, 30% of the patients in the “sitting” health state of the nusinersen arm are assumed to drop in milestones. Table A23 presents the results for this scenario analysis.

##### Table A23. Assuming 30% of Patients in the “Sitting” Lose Milestone at the End of the Short-Term Model

|  | **Treatment Costs** | **Non-Treatment Health Care Costs** | **Total Costs** | **QALYs** | **LYs** | **Incremental Results** | |
| --- | --- | --- | --- | --- | --- | --- | --- |
|  |  |  |  |  |  | **Cost/QALY Gained** | **Cost/LY Gained** |
| **nusinersen** | $1,879,000 | $1,651,000 | $3,530,000 | 2.70 | 6.95 | $1,218,000 | $601,000 |
| **BSC** | -- | $789,000 | $789,000 | 0.46 | 2.40 | -- | -- |

#### Pessimistic Scenario Analysis Assuming 30% Loss of Milestones in “Sitting” Health State and Assuming Lower Survival and Utility in Sitting Health State

Given the lack of long-term follow up and the optimistic assumptions used in the base-case analysis, we also conducted a “pessimistic scenario,” which assumes 30% of patients in the “sitting” health state lose milestones as well as lower survival and utilities for those in the “sitting” health states. Table A24 presents the results for this scenario analysis. Note that this pessimistic scenario still includes the utility benefit in the treatment arms for achieving interim milestones.

##### Table A24. Pessimistic Scenario assuming 30% of Patients in the “Sitting” Health State Lose Milestone at the End of the Short-Term Model and Assuming Lower Utilities and Lower Survival for “Sitting” Health State

|  | **Treatment Costs** | **Non-Treatment Health Care Costs** | **Total Costs** | **QALYs** | **LYs** | **Incremental Results** | |
| --- | --- | --- | --- | --- | --- | --- | --- |
|  |  |  |  |  |  | **Cost/QALY Gained** | **Cost/LY Gained** |
| **nusinersen** | $1,582,000 | $1,589,000 | $3,171,000 | 2.03 | 6.18 | $1,509,000 | $630,000 |
| **BSC** | -- | $789,000 | $789,000 | 0.46 | 2.40 | -- | -- |

#### Scenario Analysis Using 10-Year Time Horizon

Table A25 presents the results for the scenario analysis using a 10-year time horizon.

##### Table A25. Scenario analysis using a 10-Year Time Horizon

|  | **Treatment Costs** | **Non-Treatment Health Care Costs** | **Total Costs** | **QALYs** | **LYs** | **Incremental Results** | |
| --- | --- | --- | --- | --- | --- | --- | --- |
|  |  |  |  |  |  | **Cost/QALY Gained** | **Cost/LY Gained** |
| **nusinersen** | $1,484,000 | $1,338,000 | $2,822,000 | 1.85 | 5.21 | $1,460,000 | $700,000 |
| **BSC** | -- | $727,000 | $727,000 | 0.42 | 2.21 | -- | -- |

#### Scenario Analysis Using 1.5% Discounting for Costs and QALYs

Table A26 presents the results for the scenario analysis using 1.5% discounting for both costs and QALYs.

##### Table A26. Scenario analysis using 1.5% Discounting

|  | **Treatment Costs** | **Non-Treatment Health Care Costs** | **Total Costs** | **QALYs** | **LYs** | **Incremental Results** | |
| --- | --- | --- | --- | --- | --- | --- | --- |
|  |  |  |  |  |  | **Cost/QALY Gained** | **Cost/LY Gained** |
| **nusinersen** | $2,549,000 | $1,818,000 | $4,368,000 | 3.84 | 8.77 | $1,052,000 | $566,000 |
| **BSC** | -- | $834,000 | $834,000 | 0.48 | 2.53 | -- | -- |

**References**

1. Castro D, et al. Longer‐term Assessment of the Safety and Efficacy of Nusinersen for the Treatment of Infantile‐Onset Spinal Muscular Atrophy (SMA): An Interim Analysis of the SHINE Study. Paper presented at: Presented at AAN2018; Los Angeles, CA.

2. Guyot P, Ades A, Ouwens MJ, Welton NJ. Enhanced secondary analysis of survival data: reconstructing the data from published Kaplan-Meier survival curves. *BMC Medical Research Methodology.* 2012;12(1):9.

3. Finkel RS, Mercuri E, Darras BT, et al. Nusinersen versus Sham Control in Infantile-Onset Spinal Muscular Atrophy. *N Engl J Med.* 2017;377(18):1723-1732.

4. Gregoretti C, Ottonello G, Testa MBC, et al. Survival of patients with spinal muscular atrophy type 1. *Pediatrics.* 2013:2012-2278.

5. Zerres K, Rudnik-Schöneborn S, Forrest E, Lusakowska A, Borkowska J, Hausmanowa-Petrusewicz I. A collaborative study on the natural history of childhood and juvenile onset proximal spinal muscular atrophy (type II and III SMA): 569 patients. *Journal of the neurological sciences.* 1997;146(1):67-72.

6. United States Mortality Database. Human Mortality Database. 2017; <https://usa.mortality.org/>.

7. Physician Fee Schedule Search. 2018. <https://www.cms.gov/apps/physician-fee-schedule/search/search-criteria.aspx>. Accessed Nov 2nd, 2018.

8. Nationwide Children's Hospital. Price Information List. https ://<www.nationwidechildrens.org/price-information-list>. Accessed 12/19/2018.

9. Shieh PB, Gu T, Chen E. Treatment patterns and cost of care among patients with spinal muscular atrophy. SMA; 2017; Orlando.

10. Noyes J. Health and quality of life of ventilator-dependent children. *J Adv Nurs.* 2006;56(4):392-403.

11. OECD Data. OECD National Accounts Statistics: PPPs and exchange rates. 2018; <https://data.oecd.org/conversion/exchange-rates.htm>. Accessed Dec 1st, 2018.

12. Armstrong EP, Malone DC, Yeh W-S, Dahl GJ, Lee RL, Sicignano N. The economic burden of spinal muscular atrophy. *Journal of medical economics.* 2016;19(8):822-826.

13. The Lewin Group Inc. Cost of Amyotrophic Lateral Sclerosis, Muscular Dystrophy, and Spinal Muscular Atrophy in the United States. 2012; <https://www.mda.org/sites/default/files/Cost_Illness_Report_0.pdf>.

14. Bureau of Labor Statistics. Current Population Survey. 2017; <https://www.bls.gov/careeroutlook/2018/data-on-display/education-pays.htm>. Accessed 12/5/2018.

15. Lopez-Bastida J, Pena-Longobardo LM, Aranda-Reneo I, Tizzano E, Sefton M, Oliva-Moreno J. Social/economic costs and health-related quality of life in patients with spinal muscular atrophy (SMA) in Spain. *Orphanet J Rare Dis.* 2017;12(1):141.
